# Emergency Resection of a Large Third Ventricle Colloid Cyst: Ipsilateral vs. Contralateral Interhemispheric Approach Based on the Hydrocephalus Status

**DOI:** 10.7759/cureus.63960

**Published:** 2024-07-06

**Authors:** Wamedh E Matti, Hussain J Kadhum, Maher K Mustafa, Ahmed M Taha, Mustafa Ismail

**Affiliations:** 1 Department of Neurosurgery, Neurosurgery Teaching Hospital, Baghdad, IRQ; 2 Department of Neurosurgery, Fallujah Teaching Hospital, Anbar, IRQ; 3 Department of Surgery, University of Baghdad, Baghdad, IRQ

**Keywords:** surgical intervention, fainting attacks, hydrocephalus, third ventricle, colloid cyst

## Abstract

Colloid cysts of the third ventricle are rare, benign intracranial tumors that can cause significant neurological symptoms and complications, particularly when they lead to obstructive hydrocephalus. The aim of this study is to present a case of a large third ventricle colloid cyst causing acute hydrocephalus and fainting attacks, necessitating emergency surgery. This is a case of a 46-year-old female presenting with headaches and recurrent fainting attacks. Cardiac evaluations were normal. Brain MRI revealed a 3x3 cm cystic lesion in the anterior superior portion of the third ventricle, causing moderate hydrocephalus with a transependymal edema. Due to acute hydrocephalus and fainting attacks attributed to arrhythmias from hypothalamic compression, emergency surgical resection was performed. A contralateral interhemispheric transcallosal approach with a right frontal craniotomy was used to achieve gross total resection. Postoperative recovery was uneventful, and a follow-up MRI showed an empty tumor bed and resolved hydrocephalus. In conclusion, prompt diagnosis and emergency surgical intervention are crucial in cases of acute hydrocephalus caused by third ventricle colloid cysts. The successful outcome of this emergency resection demonstrates the effectiveness of timely surgical management in preventing severe complications.

## Introduction

Colloid cysts of the third ventricle are rare, benign intracranial tumors that can cause significant neurological symptoms due to their location and potential to obstruct cerebrospinal fluid pathways, leading to hydrocephalus. They often present with non-specific symptoms, including headaches and fainting attacks [1-3]. The aim of this study is to present a unique case of a large third ventricle colloid cyst causing hydrocephalus and fainting attacks. This case required emergency resection with a contralateral interhemispheric approach due to the acute hydrocephalus and recurrent fainting attacks caused by arrhythmias. Although colloid cysts can often be managed electively, the patient's acute condition necessitated immediate surgical intervention as part of our emergency surgeries to prevent further deterioration and potentially fatal complications.

## Case presentation

**Figure 1:**
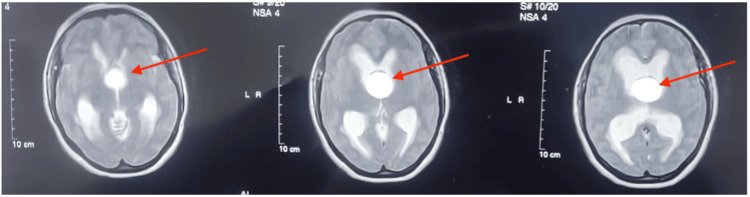
MRI images showing axial T2-weighted sections of the brain There is a large, well-defined, hyperintense cystic lesion (red arrow) located in the anterior superior portion of the third ventricle. The lesion measures approximately 3x3 cm. The surrounding brain structures indicate the presence of moderate hydrocephalus, evidenced by the dilation of the lateral ventricles and transependymal edema. The cerebrospinal fluid appears to be obstructed by the cyst, contributing to the enlargement of the ventricles. The anatomical details suggest that the lesion is consistent with a colloid cyst, though differential diagnoses such as an epidermoid cyst were considered.

**Figure 2:**
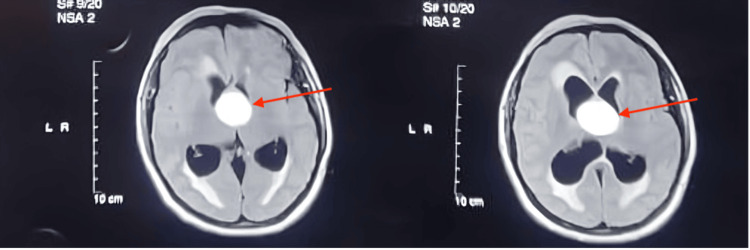
MRI images showing axial T2 FLAIR sections of the brain There is a well-defined, hyperintense cystic lesion (red arrow) located in the anterior superior portion of the third ventricle, measuring approximately 3x3 cm with hydrocephalus FLAIR - fluid-attenuated inversion recovery

**Figure 3:**
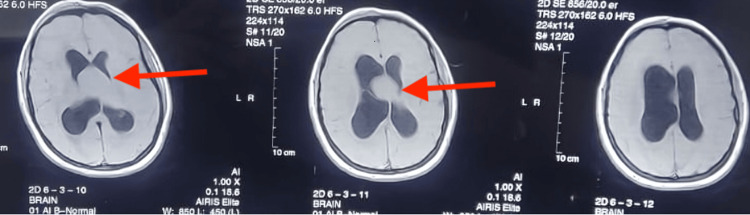
MRI images showing axial T1 sections of the brain There is a well-defined, hyperintense cystic lesion (red arrow) in the third ventricle with hydrocephalus

A 46-year-old female presented with a persistent headache and recurrent fainting attacks. Initial cardiac evaluations, including ECG and echocardiography, were normal. Brain MRI revealed a 3x3 cm round, well-defined cystic lesion (low to iso intense in T1, hyperintense in T2 and fluid-attenuated inversion recovery (FLAIR)) in the anterior superior portion of the third ventricle, with moderate hydrocephalus of the lateral ventricles (Figures 1, 2, 3). The differential diagnoses included a colloid cyst and an epidermoid cyst. The MRI also indicated a transependymal edema, suggestive of cerebrospinal fluid obstruction.

Due to the acute hydrocephalus and the patient's recurrent fainting attacks, which were hypothesized to result from arrhythmias caused by hypothalamic compression, an emergency resection of the cyst was deemed necessary. This decision was made despite the fact that colloid cysts are often managed electively, as the patient's acute condition required immediate intervention to prevent further deterioration.

**Figure 4:**
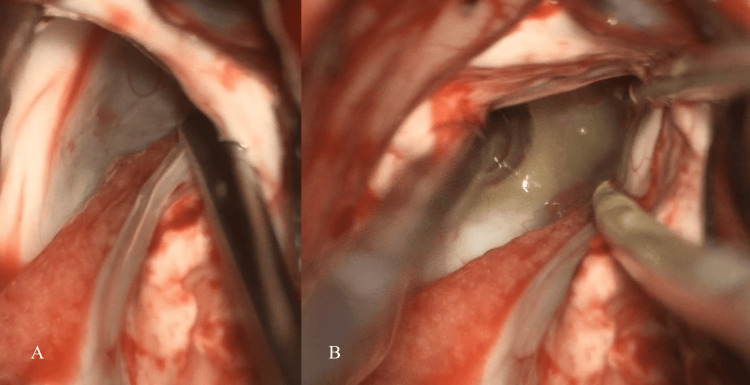
Intraoperative images of interhemispheric transcallosal approach (A) The wall of the colloid cyst is seen in the left foramen of Monro; (B) fluid inside the colloid cyst

**Figure 5:**
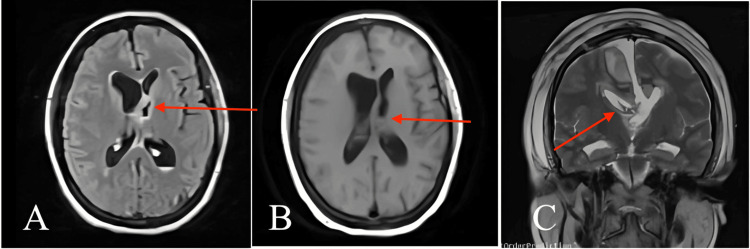
Postoperative MRI of the brain (A) axial T2 FLAIR showed gross total resection of the cyst (red arrow); (B) axial T1 showed no evidence of residual tumor or cyst (red arrow); (C) coronal T2 image shows the directory of the surgical approach and confirms the gross total resection and delineate the cyst cavity (red arrow). FLAIR - fluid-attenuated inversion recovery

An interhemispheric transcallosal approach was adopted for the surgery. Specifically, the contralateral left lateral ventricle was used to approach the contralateral left foramen of Monro through a right frontal craniotomy and interhemispheric approach. Gross total resection of the cyst was successfully performed (Figure 4). The patient's postoperative course was uneventful. A follow-up MRI showed an empty tumor bed and resolution of the hydrocephalus (Figure 5). This case highlights the importance of timely surgical intervention in cases of acute hydrocephalus caused by colloid cysts to prevent potentially fatal complications.

## Discussion

Colloid cysts of the third ventricle, while benign, can lead to severe complications if not promptly diagnosed and treated. They often present with symptoms such as headache, nausea, and in severe cases, fainting or sudden neurological deterioration due to acute hydrocephalus [1,2]. In this case, the patient's symptoms of headache and fainting were directly attributable to the cystic lesion obstructing the cerebrospinal fluid pathways, leading to hydrocephalus.

The patient's fainting attacks were hypothesized to result from hypothalamic compression by the space-occupying lesion in the third ventricle. The hypothalamus, being in close proximity to the third ventricle, can be affected by the mass effect of the cyst, potentially leading to dysregulation of autonomic functions. This compression can cause arrhythmias, which might explain the fainting episodes observed in this patient. Similar mechanisms have been suggested in the literature, highlighting the critical role of the hypothalamus in maintaining cardiovascular stability [1,2,4].

Due to the acute hydrocephalus and the patient's recurrent fainting attacks, which were caused by arrhythmias, an emergency resection of the cyst was necessary. Although colloid cysts can often be managed electively, this patient's acute condition required immediate surgical intervention as part of our emergency surgeries to prevent further deterioration.

The choice of the interhemispheric transcallosal approach allowed for optimal access to the third ventricle and facilitated a gross total resection of the cyst. Specifically, the contralateral left lateral ventricle was used to approach the contralateral left foramen of Monro through a right frontal craniotomy and interhemispheric approach. This surgical method is preferred for its ability to minimize brain manipulation and reduce the risk of postoperative complications. In cases where the ipsilateral approach is used in the presence of a trapped hydrocephalic frontal horn, there is a higher risk of cingulate gyrus lacerations. Therefore, the decision between an ipsilateral and contralateral interhemispheric approach is carefully made based on the hydrocephalus status to ensure effective treatment while minimizing brain injury and postoperative risks [5]. Postoperative imaging confirmed the effectiveness of the surgery, showing an empty tumor bed and no signs of residual hydrocephalus.

This case underscores the importance of considering a third ventricle colloid cyst in patients presenting with unexplained headaches and fainting spells, especially when accompanied by imaging findings suggestive of hydrocephalus. Early diagnosis and timely surgical intervention are crucial to prevent potentially fatal complications such as acute obstructive hydrocephalus and sudden death [3,4].

The case also highlights the varied clinical presentations of colloid cysts, reinforcing the need for a high index of suspicion and comprehensive diagnostic imaging to ensure accurate diagnosis and appropriate management [6]. The contralateral interhemispheric approach can limit the amount of brain manipulation and the likelihood of postoperative problems; this surgical technique is currently the method of choice in such cases. The successful outcome in this patient, with resolution of symptoms and no postoperative complications, exemplifies the effectiveness of current surgical techniques in managing this rare but potentially dangerous condition.

## Conclusions

This case highlights the critical need for timely diagnosis and intervention in patients with third ventricle colloid cysts, especially when presenting with acute hydrocephalus and recurrent fainting attacks caused by arrhythmias. Although these tumors are often managed electively, the patient's acute condition necessitated an emergency resection to prevent further deterioration and potentially fatal complications. In the emergency resection of a large third ventricle colloid cyst, the choice between an ipsilateral and contralateral interhemispheric approach is determined by the hydrocephalus status. An ipsilateral approach may require greater retraction of the cingulate gyrus, increasing the risk of brain injury, while a contralateral approach can minimize brain retraction and reduce potential damage. The approach selection hinges on the extent and side of ventricular dilation to ensure effective treatment with minimal brain injury. The successful surgical outcome, with a resolution of symptoms and no postoperative complications, underscores the importance of emergency surgeries in such critical cases to ensure patient safety and recovery.
